# The calcium awakens: new insights in cardiac gene therapy

**DOI:** 10.3389/fendo.2025.1738693

**Published:** 2025-12-10

**Authors:** Gaetano Santulli

**Affiliations:** 1 International Translational Research and Medical Education (ITME) Consortium, Joint Academic Research Unit, Department of Advanced Biomedical Sciences, “Federico II” University, Naples, Italy; 2 Wilf Family Cardiovascular Research Institute, Albert Einstein College of Medicine, New York, NY, United States

**Keywords:** AAV, Ca^2+^, clinical trial, gene therapy, heart failure, personalized medicine, phosphatase inhibition, SERCA2a

## Abstract

Heart failure continues to impose a major global burden, with limited options for reversing progressive contractile dysfunction despite optimized pharmacologic and device therapy. In this context, the first-in-human trial of AB-1002, a cardiotropic adeno-associated viral (AAV) vector encoding a constitutively active form of protein phosphatase-1 inhibitor (I-1c) represents a major innovation. By releasing SERCA2a from phospholamban-mediated inhibition, this strategy seeks to restore calcium cycling and contractile reserve without introducing exogenous pump proteins. In an open-label phase 1 study of 11 patients with advanced nonischemic cardiomyopathy, intracoronary delivery of AB-1002 was well tolerated, with no serious vector-related adverse events and only mild transient hepatic enzyme elevations. Modest but consistent improvements were observed in LVEF, while myocardial tissue from one explanted heart confirmed successful transgene expression and phospholamban phosphorylation. These results demonstrate the feasibility and biological activity of a phosphatase-inhibition gene-therapy approach for human heart failure. The forthcoming phase 2 GenePHIT trial will determine whether these encouraging mechanistic signals can be translated into tangible clinical benefit. AB-1002 thus represents a cautiously optimistic inflection point—suggesting that, with improved vector design and rigorous evaluation, gene therapy may yet deliver on its long-sought promise of molecular restoration in the failing human heart.

## Introduction

Heart failure (HF) remains one of the most intractable syndromes in modern medicine (1). Despite decades of pharmacological progress and remarkable advances in device therapy, the clinical trajectory for patients with severe systolic dysfunction often converges on decline and death (2, 3). A recent trial by Henry and colleagues, published in *Nature Medicine*, reports the first-in-human use of AB-1002, a cardiotropic adeno-associated viral (AAV) vector designed to deliver a constitutively active form of the protein phosphatase 1 inhibitor (I-1c) directly into the myocardium (4). The findings—demonstrating feasibility, apparent biological activity, and encouraging safety in a small cohort of patients with advanced nonischemic cardiomyopathy—represent both a milestone and a reminder of how complex the path to cardiac gene therapy remains.

## A heart in need of new solutions

Globally, more than 60 million people live with HF, half with reduced left ventricular ejection fraction (LVEF). Even with the full complement of contemporary medical therapy—including β-blockers, renin-angiotensin-system antagonists, mineralocorticoid receptor antagonists, ARNI, and SGLT2 inhibitors—many patients progress inexorably to advanced disease (2). The molecular and cellular mechanisms of contractile failure are well established: altered Ca^2+^ cycling, β-adrenergic desensitization, sarcomeric dysfunction, and maladaptive remodeling culminate in a myocardium unable to generate adequate force (5–7). Therapeutic strategies pharmacologically targeting these pathways have achieved only partial success (2). The concept of directly reprogramming the molecular machinery of contraction through gene transfer has therefore persisted as a tantalizing possibility (**Table 1**).

**Table 1:** Chronological perspective of the main clinical trials testing gene therapy in cardiovascular disorders.

Years	Trial	Target	Vector	Indication	Main findings	Refs
Late 1990s–early 2000s	VEGF & FGF plasmid/adenoviral programs (e.g., VIVA, Kuopio, Euroinject-One, KAT)	VEGF165/FGF (angiogenic growth factors)	Plasmid DNA or Ad vectors; intramyocardial/intracoronary	Refractory angina, ischemic heart disease	Early studies showed transient perfusion improvement; no mortality benefit. Pioneered catheter/surgical injection approaches.	(22, 23)
1999–2004	BIOBYPASS/Ad.GVVEGF121 (NOVA)	VEGF121	Adenovirus; intramyocardial injection	Refractory angina	Safe; some improvement in perfusion and angina class; not durable in larger trials.	(24)
Early 2000s	AdVEGF121/MAGIC/AGENT-1/2	VEGF121/FGF4	Adenoviral vectors; intracoronary	Stable/refractory angina	Demonstrated feasibility and biologic activity; led to larger AGENT trials.	(25, 26)
2003–2007	AGENT-3 & AGENT-4 (Ad5FGF4)	FGF-4	Adenovirus 5; intracoronary	Refractory angina/ischemic heart disease	Large RCTs terminated for futility; no efficacy benefit.	(27)
2005–2008	CUPID-1 (AAV1-SERCA2a)	SERCA2a	AAV1; intracoronary infusion	Advanced HFrEF	Showed safety and promising biomarker/functional improvements.	(8, 9)
2009–2013	CUPID-2 (AAV1-SERCA2a; NCT01643330)	SERCA2a	AAV1; intracoronary	HFrEF	No clinical benefit; dashed early hopes for cardiac gene therapy.	(10)
2010–2012	Neovasculgen (Pl-VEGF165; NCT02538705)	VEGF165	Plasmid DNA; intramuscular	Critical limb ischemia/PAD	Improved perfusion indices; approved in Russia (first regional cardiovascular gene therapy).	(28, 29)
2012–2015	Ad5.hAC6 (Adenylyl Cyclase 6)	AC6	Adenovirus-5; intracoronary infusion	HF with reduced EF	Improved LV function and cAMP signalling; safe.	(30)
2020–2023	AB-1002 (AAV2i8-I-1c; NCT04179643)	Constitutively active I-1 (PP1 inhibitor)	AAV2/AAV8 hybrid; intracoronary antegrade	Nonischemic HFrEF (NYHA III)	Safe and biologically active; improved LVEF/NYHA; forms basis for phase 2 GenePHIT.	(4)
2023–ongoing	GenePHIT (NCT05598333)	I-1c (same as AB-1002)	AAV2i8; intracoronary	Nonischemic HFrEF	Recruiting; evaluates two doses, ~150 patients, includes AAV-seropositive individuals.	–
Emerging (2024 → ongoing)	Pulmonary vascular/BMPR2 restoration programs	BMPR2 or other PAH targets	AAV/mRNA/nanoparticle	Pulmonary arterial hypertension	Translational stage; aims to restore BMPR2 signaling.	–

Previous attempts to achieve this goal, most notably through AAV-mediated expression of SERCA2a in the CUPID (Calcium Upregulation by Percutaneous Administration of Gene Therapy in Cardiac Disease) trials (8–10), established the safety of cardiac gene transfer but fell short of demonstrating durable efficacy. The failure of CUPID-2 tempered enthusiasm and underscored the formidable biological and logistical barriers to gene therapy for HF (10): vector tropism, transduction efficiency, immune responses, and the complexity of the failing human myocardium itself. Yet these setbacks also refined the field’s understanding of what must change for success—chiefly, the need for more selective, cardiotropic vectors and for molecular targets that act upstream of SERCA2a expression.

## Mechanistic innovation: targeting phosphatase inhibition

The innovative approach pursued by the team led by Roger Hajjar (4) lies in restoring Ca^2+^ homeostasis not by overexpressing SERCA2a but by releasing it from inhibition. In the failing heart, hyperactivation of protein phosphatase 1 (PP1) leads to dephosphorylation of phospholamban (PLN), which suppresses SERCA2a activity and slows Ca^2+^ reuptake into the sarcoplasmic reticulum (11). The endogenous inhibitor of PP1, I-1, is hypoactive in HF; its constitutively active form (I-1c) can, in theory, restore PLN phosphorylation, enhance Ca^2+^ cycling, and improve contractility without overloading the cardiomyocyte with exogenous proteins (12).

AB-1002 encapsulates this concept in a chimeric AAV2/AAV8 vector (AAV2i8) optimized for cardiac tropism and reduced hepatic uptake. By delivering I-1c locally through intracoronary infusion, the therapy aims to maximize myocardial transduction while minimizing systemic exposure (13).

This mechanistic precision reflects lessons learned from earlier gene therapy efforts. The use of a non-integrating vector reduces genotoxic risk, while the cardiotropic capsid offers a higher likelihood of transduction in human myocardium, a historical weakness of AAV1-based constructs (14). Furthermore, intracoronary infusion—though technically demanding—allows localized dosing within a controlled procedure familiar to interventional cardiologists.

### Findings that hint at promise

This phase 1 trial enrolled 11 patients with New York Heart Association (NYHA) class III nonischemic cardiomyopathy and LVEF between 15% and 35%; two dose levels were tested: 3.25 × 10^13^ and 1.08 × 10^14^ viral genomes (4). The results, while preliminary, are notable for what they do not show: there were no treatment-related serious adverse events, no evidence of myocarditis or systemic toxicity, and only mild, transient elevations of liver enzymes at the higher dose. The absence of significant inflammatory sequelae—particularly in patients unable to receive prophylactic corticosteroids because of the risk of worsening myopathy—suggests a favorable immunological profile of this vector–transgene combination.

Clinically, modest but consistent improvements were observed across several measures. Most patients experienced an improvement of at least one NYHA class. Strikingly, LVEF rose from a mean of 30% to 42% in the lower-dose cohort and from 25% to 37% in the higher-dose group. Functional parameters such as six-minute walk distance and quality-of-life scores also trended positively, though heterogeneously (4). One patient who later underwent left ventricular assist device (LVAD) implantation displayed robust myocardial transduction and normalization of PLN phosphorylation levels compared to those seen in healthy controls—direct evidence of biological activity at the tissue level.

While these results must be interpreted cautiously, the study provides the first demonstration that a PP1-inhibition strategy can be implemented safely in humans using a cardiac-specific AAV platform. For a field that has long wrestled with delivery and toxicity hurdles, this is no small achievement.

## Discussion

### A cautious revival of cardiac gene therapy

The most compelling aspect of this trial may not be its measured efficacy signals but its reinvigoration of a field long in search of vindication. Since the disappointing results of CUPID-2 nearly a decade ago (10), cardiac gene therapy has been overshadowed by the meteoric success of AAV-mediated treatments in neuromuscular and metabolic disorders (15). The re-emergence of a cardiotropic vector capable of safely transducing human myocardium rekindles the possibility that gene therapy could finally move from aspiration to reality in HF.

That said, the broader landscape has changed. The standard of care now includes therapies with proven survival benefits (16), leaving gene therapy to justify its role either as an adjunct in refractory disease or as a disease-modifying intervention in earlier stages. To compete, gene therapy must demonstrate not only safety but durable efficacy, reproducibility, and economic feasibility (17).

The next phase 2 study, GenePHIT (NCT05598333), aims to enroll up to 150 patients using two doses that straddle the apparent therapeutic window observed here. It will include patients with preexisting neutralizing antibodies, an important test of real-world applicability. Yet, we must acknowledge that translating molecular correction into clinical improvement will demand more than vector optimization—it will require a better understanding of how much functional myocardium remains amenable to rescue in advanced disease and whether restoring Ca^2+^ cycling can truly reverse structural remodeling.

### Lessons in translation

This study also highlights enduring translational lessons. First, vector tropism matters. The AAV2i8 capsid’s enhanced myocardial affinity and reduced hepatic uptake are clear advantages over earlier serotypes. Even with such engineering, human transduction efficiency remains orders of magnitude below that achieved in animal models (18), underscoring the limits of preclinical predictability.

Second, the target matters. By focusing on I-1c, Henry and colleagues are exploiting a nodal regulator of cardiac contractility that integrates multiple upstream pathways. However, PP1 activity extends beyond Ca^2+^ handling to diverse cellular processes (19); sustained inhibition could theoretically predispose to arrhythmias, hypertrophy, or maladaptive signaling. Long-term follow-up will be essential to ensure that apparent benefits do not mask latent toxicity.

Third, patient selection is crucial. The participants in this trial had advanced non-ischemic cardiomyopathy with low LVEF, yet one might speculate that gene therapy would yield greater benefit in earlier disease, before irreversible fibrosis and myocyte loss. Balancing the ethical imperative to protect the sickest patients with the biological rationale to treat earlier will be a recurring challenge as the field matures.

Finally, trial design must evolve beyond the traditional paradigms of small open-label safety studies. Adaptive designs, surrogate-to-outcome linkages, and the integration of cardiac imaging and molecular biomarkers could accelerate progress while maintaining rigor. Gene therapy trials also demand transparent, independent safety monitoring to sustain public confidence—particularly as the boundaries between academic innovation and commercial development blur.

### Limitations

The enthusiasm must be tempered by the realities of the study’s design. This was an open-label, single-arm phase 1 trial with only 11 patients, without independent safety oversight or a predefined dose-limiting toxicity window. The lack of a control group precludes any firm conclusion about efficacy, and the observed improvements could reflect regression to the mean, natural variability, or changes in concomitant medical therapy. The two cohorts differed substantially in age, baseline LVEF, and exercise capacity—imbalances that further complicate interpretation.

The immunologic findings, although manageable, deserve scrutiny. The transient T-cell responses directed against both the AAV capsid and the I-1c transgene indicate that cellular immunity remains a persistent obstacle to durable transgene expression. Mild elevations in alanine and aspartate aminotransferases, temporally linked to interferon-γ responses, suggest low-grade hepatic involvement even with reduced liver tropism (20). Future studies must clarify whether these immune phenomena compromise long-term vector persistence or limit redosing potential—an especially relevant issue given that baseline neutralizing antibodies excluded several participants.

The absence of a clear dose–response relationship also raises questions. Functional gains appeared similar or even greater in the lower-dose cohort, prompting investigators to forgo escalation to the highest planned dose. This finding could indicate a narrow therapeutic window, or alternatively, a plateau of biological effect at lower vector copy numbers. Either interpretation has implications for the scalability and cost-effectiveness of any eventual therapy.

Methodologically, the reliance on surrogate markers—LVEF, six-minute walk test, peak VO_2_—rather than hard clinical outcomes is appropriate for phase 1 safety evaluation but insufficient to establish clinical benefit. The heterogeneity and the limited follow-up (12 months) of the trial make it impossible to infer effects on hospitalization or survival. Moreover, changes in quality-of-life scores, while directionally favorable, were modest and subject to substantial variability.

### The path forward

If AB-1002 ultimately succeeds, it will do so not simply because it improves LVEF, but because it proves that the human heart can be durably reprogrammed by genetic means. Such a demonstration would open a therapeutic era in which cardiomyocytes are not merely supported but molecularly corrected. However, even a positive phase 2 result will leave unanswered questions about vector persistence, immune memory, manufacturing scalability, and cost. The eventual integration of gene therapy into heart-failure management will require partnerships among academic investigators, regulatory agencies, and industry to ensure equitable access and long-term safety surveillance.

Ethically, the deployment of irreversible genetic interventions in nonlethal chronic disease warrants special scrutiny. Unlike one-time curative gene therapies for monogenic disorders, cardiac gene therapy targets a multifactorial condition that remains responsive to conventional therapy (21). Determining the appropriate threshold for intervention—and ensuring informed consent that reflects both the promise and the uncertainty—will be paramount.

### A *cautiously* optimistic outlook

In sum, the phase 1 trial of AB-1002 marks a measured but meaningful advance in the long pursuit of molecular restoration for the failing heart. The safety profile, mechanistic clarity, and early efficacy signals justify cautious optimism. The study also underscores the fragility of translational momentum in this space: without rigorous, controlled, and sufficiently powered trials, the field risks repeating the cycle of premature enthusiasm followed by disillusionment.

Still, the concept of selectively modulating phosphatase activity to recalibrate Ca^2+^ cycling is intellectually elegant and biologically grounded. Should these findings be confirmed in larger studies, AB-1002—or its successors—could inaugurate a new therapeutic class: precision gene therapies for acquired cardiovascular disease. For now, this work invites renewed attention to the question that has animated cardiac gene therapy for two decades: can we teach a failing human heart to remember how to beat normally again? The answer, for the first time in years, feels within reach.

## Data Availability

The original contributions presented in the study are included in the article/supplementary material. Further inquiries can be directed to the corresponding author/s.
